# 2-(3,5-Di-*tert*-butyl-4-hydroxy­benzyl­sulfan­yl)-*N*′-(3-methoxy­benzyl­idene)acetohydrazide

**DOI:** 10.1107/S1600536809030645

**Published:** 2009-08-08

**Authors:** Wagee A. Yehye, Azhar Ariffin, Noorsaadah Abdul Rahman, Seik Weng Ng

**Affiliations:** a Department of Chemistry, University of Malaya, 50603 Kuala Lumpur, Malaysia

## Abstract

The title compound, C_25_H_34_N_2_O_3_S, is a derivative of *N*′-benzyl­ideneacetohydrazide having substituents on the acetyl and benzylidenyl parts, and displays a planar C_carbon­yl_—NH—NC_anis­yl_ fragment [torsion angle = 174.9 (3)°]. The –NH– unit forms an N—H⋯O hydrogen bond with the carbonyl O atom of an inversion-related mol­ecule.

## Related literature

For *N*-(benzyl­idene)acetohydrazide, see: Litvinov *et al.* (1991 ▶).
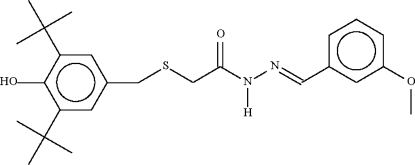


## Experimental

### 

#### Crystal data

C_25_H_34_N_2_O_3_S
*M* *_r_* = 442.60
Triclinic, 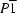

*a* = 5.9952 (2) Å
*b* = 10.3199 (3) Å
*c* = 20.6141 (7) Å
α = 97.279 (2)°
β = 95.916 (2)°
γ = 99.981 (2)°
*V* = 1235.72 (7) Å^3^
*Z* = 2
Mo *K*α radiation
μ = 0.16 mm^−1^
*T* = 223 K
0.35 × 0.10 × 0.10 mm

#### Data collection

Bruker SMART APEX diffractometer
Absorption correction: multi-scan (*SADABS*; Sheldrick, 1996 ▶) *T*_min_ = 0.947, *T*_max_ = 0.984
8285 measured reflections
4294 independent reflections
2577 reflections with *I* > 2σ(*I*)
*R*_int_ = 0.035

#### Refinement

*R*[*F* ^2^ > 2σ(*F* ^2^)] = 0.075
*wR*(*F* ^2^) = 0.219
*S* = 1.06
4294 reflections
287 parameters
H-atom parameters constrained
Δρ_max_ = 0.61 e Å^−3^
Δρ_min_ = −0.27 e Å^−3^

### 

Data collection: *APEX2* (Bruker, 2008 ▶); cell refinement: *SAINT* (Bruker, 2008 ▶); data reduction: *SAINT*; program(s) used to solve structure: *SHELXS97* (Sheldrick, 2008 ▶); program(s) used to refine structure: *SHELXL97* (Sheldrick, 2008 ▶); molecular graphics: *X-SEED* (Barbour, 2001 ▶); software used to prepare material for publication: *publCIF* (Westrip, 2009 ▶).

## Supplementary Material

Crystal structure: contains datablocks global, I. DOI: 10.1107/S1600536809030645/xu2573sup1.cif

Structure factors: contains datablocks I. DOI: 10.1107/S1600536809030645/xu2573Isup2.hkl

Additional supplementary materials:  crystallographic information; 3D view; checkCIF report

## Figures and Tables

**Table 1:** Hydrogen-bond geometry (Å, °)

*D*—H⋯*A*	*D*—H	H⋯*A*	*D*⋯*A*	*D*—H⋯*A*
N1—H1⋯O2^i^	0.88	2.00	2.884 (4)	176

Symmetry code: (i) 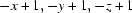.

**Fig. 1.**
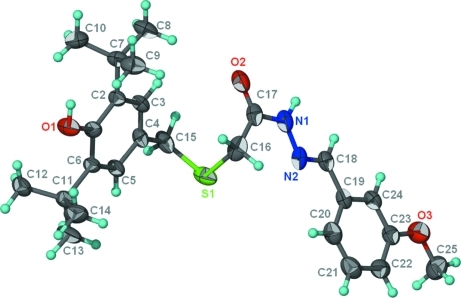
Thermal ellipsoid plot (Barbour, 2001) of C25H34N2O3S at the 70% probability level; hydrogen atoms are drawn as spheres of arbitrary radius.

## supplementary crystallographic information

### Experimental

2-(3,5-Di-*tert*-butyl-4-hydroxybenzylthio)acetohydrazine (0.5 g, 1.54 mmol) and 3-methoxybenzaldehyde (0.21 g, 1.54 mmol) were heated in toluene (10 ml) for 4 h in the presence of *p*-toluene sulphonic acid as catalyst. The cool mixture was poured onto ice; the resulting solid was collected, dried and recrystallized from toluene in 70% yield.

### Refinement

Carbon-bound H-atoms were placed in calculated positions (C—H 0.95 to 0.99 Å) and were included in the refinement in the riding model approximation, with *U*(H) set to 1.2 to 1.5*U*(C). The imino H-atom was similarly treated (N–H 0.88 Å).; the hydroxy H-atom was placed in a position such that all H···H contacts exceeded 2.0 Å (O–H 0.84 Å).

### Crystal data

**Table:**

C_25_H_34_N_2_O_3_S	*Z* = 2
*M**_r_* = 442.60	*F*(000) = 476
Triclinic, *P*1	*D*_x_ = 1.190 Mg m^−^^3^
Hall symbol: -P 1	Mo *K*α radiation, λ = 0.71073 Å
*a* = 5.9952 (2) Å	Cell parameters from 1620 reflections
*b* = 10.3199 (3) Å	θ = 2.6–21.7°
*c* = 20.6141 (7) Å	µ = 0.16 mm^−^^1^
α = 97.279 (2)°	*T* = 223 K
β = 95.916 (2)°	Prism, colorless
γ = 99.981 (2)°	0.35 × 0.10 × 0.10 mm
*V* = 1235.72 (7) Å^3^	

### Data collection

**Table:**

Bruker SMART APEX diffractometer	4294 independent reflections
Radiation source: fine-focus sealed tube	2577 reflections with *I* > 2σ(*I*)
graphite	*R*_int_ = 0.035
ω scans	θ_max_ = 25.0°, θ_min_ = 1.0°
Absorption correction: multi-scan (*SADABS*; Sheldrick, 1996)	*h* = −7→7
*T*_min_ = 0.947, *T*_max_ = 0.984	*k* = −12→12
8285 measured reflections	*l* = −24→23

### Refinement

**Table:**

Refinement on *F*^2^	Primary atom site location: structure-invariant direct methods
Least-squares matrix: full	Secondary atom site location: difference Fourier map
*R*[*F*^2^ > 2σ(*F*^2^)] = 0.075	Hydrogen site location: inferred from neighbouring sites
*wR*(*F*^2^) = 0.219	H-atom parameters constrained
*S* = 1.06	*w* = 1/[σ^2^(*F*_o_^2^) + (0.1218*P*)^2^] where *P* = (*F*_o_^2^ + 2*F*_c_^2^)/3
4294 reflections	(Δ/σ)_max_ = 0.001
287 parameters	Δρ_max_ = 0.61 e Å^−^^3^
0 restraints	Δρ_min_ = −0.27 e Å^−^^3^

### Special details

**Table:**

Geometry. All e.s.d.'s (except the e.s.d. in the dihedral angle between two l.s. planes) are estimated using the full covariance matrix. The cell e.s.d.'s are taken into account individually in the estimation of e.s.d.'s in distances, angles and torsion angles; correlations between e.s.d.'s in cell parameters are only used when they are defined by crystal symmetry. An approximate (isotropic) treatment of cell e.s.d.'s is used for estimating e.s.d.'s involving l.s. planes.

### Fractional atomic coordinates and isotropic or equivalent isotropic displacement parameters (Å^2^)

**Table:**

	*x*	*y*	*z*	*U*_iso_*/*U*_eq_	
S1	0.75715 (19)	0.78337 (10)	0.36105 (5)	0.0691 (4)	
O1	0.1621 (4)	0.5619 (2)	0.06464 (11)	0.0613 (8)	
H1O	0.2538	0.5093	0.0605	0.092*	
O2	0.4442 (5)	0.5039 (2)	0.40994 (12)	0.0635 (8)	
O3	1.6583 (5)	0.8943 (3)	0.72616 (13)	0.0731 (9)	
N1	0.7590 (5)	0.5928 (3)	0.48032 (14)	0.0545 (8)	
H1	0.6926	0.5660	0.5137	0.065*	
N2	0.9784 (5)	0.6642 (3)	0.49141 (14)	0.0531 (8)	
C1	0.2353 (5)	0.6087 (3)	0.13041 (16)	0.0433 (8)	
C2	0.1613 (5)	0.5357 (3)	0.17936 (15)	0.0400 (8)	
C3	0.2413 (6)	0.5907 (3)	0.24438 (16)	0.0435 (8)	
H3	0.1965	0.5425	0.2781	0.052*	
C4	0.3841 (6)	0.7132 (3)	0.26141 (15)	0.0424 (8)	
C5	0.4527 (6)	0.7824 (3)	0.21091 (16)	0.0454 (9)	
H5	0.5517	0.8654	0.2221	0.054*	
C6	0.3814 (5)	0.7343 (3)	0.14524 (16)	0.0432 (9)	
C7	−0.0028 (6)	0.3992 (3)	0.16267 (18)	0.0495 (9)	
C8	−0.0615 (7)	0.3430 (4)	0.22584 (19)	0.0628 (11)	
H8A	0.0778	0.3330	0.2516	0.094*	
H8B	−0.1623	0.2570	0.2141	0.094*	
H8C	−0.1367	0.4036	0.2517	0.094*	
C9	0.1096 (7)	0.2961 (4)	0.1244 (2)	0.0632 (11)	
H9A	0.1537	0.3275	0.0843	0.095*	
H9B	0.0023	0.2124	0.1132	0.095*	
H9C	0.2440	0.2834	0.1515	0.095*	
C10	−0.2303 (7)	0.4132 (4)	0.1249 (2)	0.0725 (13)	
H10A	−0.2018	0.4475	0.0842	0.109*	
H10B	−0.3019	0.4741	0.1518	0.109*	
H10C	−0.3307	0.3268	0.1147	0.109*	
C11	0.4605 (6)	0.8127 (4)	0.09063 (17)	0.0538 (10)	
C12	0.2542 (7)	0.8470 (4)	0.04992 (19)	0.0673 (12)	
H12A	0.1521	0.7656	0.0291	0.101*	
H12B	0.3070	0.8982	0.0163	0.101*	
H12C	0.1735	0.8989	0.0787	0.101*	
C13	0.6181 (8)	0.9471 (4)	0.1203 (2)	0.0835 (15)	
H13A	0.7536	0.9306	0.1454	0.125*	
H13B	0.5372	0.9988	0.1490	0.125*	
H13C	0.6621	0.9959	0.0849	0.125*	
C14	0.5967 (7)	0.7360 (5)	0.0464 (2)	0.0770 (14)	
H14A	0.7316	0.7210	0.0724	0.115*	
H14B	0.6424	0.7870	0.0119	0.115*	
H14C	0.5030	0.6511	0.0266	0.115*	
C15	0.4592 (7)	0.7738 (3)	0.33293 (17)	0.0567 (10)	
H15A	0.3680	0.7211	0.3607	0.068*	
H15B	0.4253	0.8637	0.3392	0.068*	
C16	0.7607 (7)	0.6107 (4)	0.36385 (18)	0.0634 (11)	
H16A	0.6844	0.5581	0.3220	0.076*	
H16B	0.9191	0.5976	0.3693	0.076*	
C17	0.6438 (7)	0.5632 (3)	0.41904 (18)	0.0519 (9)	
C18	1.0622 (6)	0.6942 (3)	0.55202 (18)	0.0484 (9)	
H18	0.9739	0.6658	0.5845	0.058*	
C19	1.2932 (6)	0.7721 (3)	0.57209 (17)	0.0456 (9)	
C20	1.4309 (7)	0.8176 (4)	0.52624 (19)	0.0574 (10)	
H20	1.3757	0.8005	0.4810	0.069*	
C21	1.6485 (8)	0.8877 (4)	0.5475 (2)	0.0653 (11)	
H21	1.7418	0.9177	0.5164	0.078*	
C22	1.7322 (7)	0.9150 (3)	0.6137 (2)	0.0547 (10)	
H22	1.8815	0.9626	0.6276	0.066*	
C23	1.5954 (7)	0.8718 (3)	0.65910 (18)	0.0519 (9)	
C24	1.3768 (6)	0.8000 (3)	0.63807 (17)	0.0475 (9)	
H24	1.2843	0.7698	0.6693	0.057*	
C25	1.8769 (8)	0.9736 (5)	0.7502 (2)	0.0793 (14)	
H25A	1.9013	0.9819	0.7979	0.119*	
H25B	1.8843	1.0612	0.7372	0.119*	
H25C	1.9940	0.9319	0.7318	0.119*	

### Atomic displacement parameters (Å^2^)

**Table:**

	*U*^11^	*U*^22^	*U*^33^	*U*^12^	*U*^13^	*U*^23^
S1	0.0859 (8)	0.0701 (7)	0.0346 (6)	−0.0270 (6)	−0.0044 (5)	0.0117 (4)
O1	0.0721 (17)	0.0705 (17)	0.0310 (14)	−0.0100 (14)	−0.0009 (12)	0.0066 (11)
O2	0.085 (2)	0.0483 (15)	0.0520 (17)	0.0036 (14)	−0.0108 (14)	0.0139 (12)
O3	0.082 (2)	0.0767 (19)	0.0480 (17)	−0.0086 (16)	−0.0075 (14)	0.0083 (13)
N1	0.070 (2)	0.0470 (17)	0.0455 (19)	0.0155 (16)	−0.0058 (16)	0.0090 (13)
N2	0.063 (2)	0.0553 (18)	0.0430 (19)	0.0242 (16)	−0.0048 (15)	0.0065 (14)
C1	0.0435 (19)	0.054 (2)	0.0297 (18)	0.0018 (16)	0.0059 (14)	0.0076 (15)
C2	0.0431 (18)	0.0427 (19)	0.0349 (19)	0.0042 (15)	0.0099 (15)	0.0092 (14)
C3	0.055 (2)	0.0402 (19)	0.038 (2)	0.0082 (16)	0.0128 (16)	0.0129 (15)
C4	0.059 (2)	0.0423 (19)	0.0268 (18)	0.0117 (16)	0.0039 (15)	0.0062 (14)
C5	0.055 (2)	0.0417 (18)	0.0359 (19)	−0.0017 (16)	−0.0001 (15)	0.0112 (14)
C6	0.0425 (19)	0.050 (2)	0.0348 (19)	−0.0036 (16)	0.0046 (15)	0.0146 (15)
C7	0.050 (2)	0.048 (2)	0.048 (2)	−0.0032 (17)	0.0141 (17)	0.0076 (16)
C8	0.080 (3)	0.049 (2)	0.060 (3)	−0.002 (2)	0.026 (2)	0.0144 (18)
C9	0.070 (3)	0.051 (2)	0.064 (3)	−0.003 (2)	0.019 (2)	−0.0008 (19)
C10	0.052 (2)	0.072 (3)	0.087 (3)	−0.010 (2)	0.005 (2)	0.019 (2)
C11	0.058 (2)	0.063 (2)	0.034 (2)	−0.0093 (19)	−0.0006 (17)	0.0154 (17)
C12	0.072 (3)	0.078 (3)	0.049 (2)	−0.004 (2)	0.000 (2)	0.033 (2)
C13	0.097 (3)	0.080 (3)	0.056 (3)	−0.039 (3)	−0.006 (2)	0.031 (2)
C14	0.064 (3)	0.111 (4)	0.054 (3)	−0.007 (3)	0.019 (2)	0.027 (2)
C15	0.091 (3)	0.0354 (19)	0.043 (2)	0.0132 (19)	0.004 (2)	0.0085 (15)
C16	0.064 (2)	0.086 (3)	0.039 (2)	0.027 (2)	0.0017 (18)	−0.0077 (19)
C17	0.070 (3)	0.042 (2)	0.043 (2)	0.0183 (19)	−0.0060 (19)	0.0027 (16)
C18	0.061 (2)	0.0413 (19)	0.046 (2)	0.0183 (17)	0.0036 (18)	0.0109 (16)
C19	0.059 (2)	0.0429 (19)	0.040 (2)	0.0186 (17)	0.0070 (17)	0.0114 (15)
C20	0.073 (3)	0.063 (2)	0.043 (2)	0.025 (2)	0.0118 (19)	0.0108 (18)
C21	0.081 (3)	0.068 (3)	0.059 (3)	0.028 (2)	0.030 (2)	0.023 (2)
C22	0.055 (2)	0.045 (2)	0.068 (3)	0.0146 (18)	0.015 (2)	0.0109 (18)
C23	0.063 (2)	0.041 (2)	0.051 (2)	0.0108 (18)	0.0031 (19)	0.0114 (17)
C24	0.060 (2)	0.0440 (19)	0.040 (2)	0.0101 (17)	0.0085 (17)	0.0112 (15)
C25	0.073 (3)	0.080 (3)	0.073 (3)	0.002 (2)	−0.019 (2)	0.005 (2)

### Geometric parameters (Å, °)

**Table:**

S1—C16	1.794 (4)	C10—H10C	0.9700
S1—C15	1.802 (4)	C11—C14	1.524 (6)
O1—C1	1.379 (4)	C11—C12	1.540 (5)
O1—H1O	0.8400	C11—C13	1.549 (5)
O2—C17	1.230 (4)	C12—H12A	0.9700
O3—C23	1.374 (4)	C12—H12B	0.9700
O3—C25	1.423 (5)	C12—H12C	0.9700
N1—C17	1.347 (4)	C13—H13A	0.9700
N1—N2	1.373 (4)	C13—H13B	0.9700
N1—H1	0.8800	C13—H13C	0.9700
N2—C18	1.276 (4)	C14—H14A	0.9700
C1—C2	1.397 (4)	C14—H14B	0.9700
C1—C6	1.411 (4)	C14—H14C	0.9700
C2—C3	1.389 (4)	C15—H15A	0.9800
C2—C7	1.548 (4)	C15—H15B	0.9800
C3—C4	1.380 (4)	C16—C17	1.487 (5)
C3—H3	0.9400	C16—H16A	0.9800
C4—C5	1.392 (4)	C16—H16B	0.9800
C4—C15	1.516 (5)	C18—C19	1.465 (5)
C5—C6	1.378 (5)	C18—H18	0.9400
C5—H5	0.9400	C19—C24	1.376 (5)
C6—C11	1.534 (4)	C19—C20	1.391 (5)
C7—C10	1.538 (5)	C20—C21	1.375 (6)
C7—C9	1.534 (5)	C20—H20	0.9400
C7—C8	1.540 (5)	C21—C22	1.382 (5)
C8—H8A	0.9700	C21—H21	0.9400
C8—H8B	0.9700	C22—C23	1.373 (5)
C8—H8C	0.9700	C22—H22	0.9400
C9—H9A	0.9700	C23—C24	1.385 (5)
C9—H9B	0.9700	C24—H24	0.9400
C9—H9C	0.9700	C25—H25A	0.9700
C10—H10A	0.9700	C25—H25B	0.9700
C10—H10B	0.9700	C25—H25C	0.9700
			
C16—S1—C15	100.04 (17)	C11—C12—H12C	109.5
C1—O1—H1O	95.8	H12A—C12—H12C	109.5
C23—O3—C25	117.4 (3)	H12B—C12—H12C	109.5
C17—N1—N2	120.9 (3)	C11—C13—H13A	109.5
C17—N1—H1	119.6	C11—C13—H13B	109.5
N2—N1—H1	119.6	H13A—C13—H13B	109.5
C18—N2—N1	114.8 (3)	C11—C13—H13C	109.5
O1—C1—C2	120.9 (3)	H13A—C13—H13C	109.5
O1—C1—C6	116.7 (3)	H13B—C13—H13C	109.5
C2—C1—C6	122.4 (3)	C11—C14—H14A	109.5
C3—C2—C1	117.1 (3)	C11—C14—H14B	109.5
C3—C2—C7	120.8 (3)	H14A—C14—H14B	109.5
C1—C2—C7	122.0 (3)	C11—C14—H14C	109.5
C4—C3—C2	122.6 (3)	H14A—C14—H14C	109.5
C4—C3—H3	118.7	H14B—C14—H14C	109.5
C2—C3—H3	118.7	C4—C15—S1	115.0 (3)
C3—C4—C5	118.1 (3)	C4—C15—H15A	108.5
C3—C4—C15	121.4 (3)	S1—C15—H15A	108.5
C5—C4—C15	120.4 (3)	C4—C15—H15B	108.5
C6—C5—C4	122.7 (3)	S1—C15—H15B	108.5
C6—C5—H5	118.7	H15A—C15—H15B	107.5
C4—C5—H5	118.7	C17—C16—S1	111.4 (3)
C5—C6—C1	117.0 (3)	C17—C16—H16A	109.4
C5—C6—C11	121.5 (3)	S1—C16—H16A	109.4
C1—C6—C11	121.5 (3)	C17—C16—H16B	109.4
C10—C7—C9	111.7 (3)	S1—C16—H16B	109.4
C10—C7—C8	106.5 (3)	H16A—C16—H16B	108.0
C9—C7—C8	105.8 (3)	O2—C17—N1	120.4 (4)
C10—C7—C2	110.6 (3)	O2—C17—C16	121.4 (3)
C9—C7—C2	110.9 (3)	N1—C17—C16	118.1 (4)
C8—C7—C2	111.1 (3)	N2—C18—C19	121.4 (3)
C7—C8—H8A	109.5	N2—C18—H18	119.3
C7—C8—H8B	109.5	C19—C18—H18	119.3
H8A—C8—H8B	109.5	C24—C19—C20	119.1 (3)
C7—C8—H8C	109.5	C24—C19—C18	119.1 (3)
H8A—C8—H8C	109.5	C20—C19—C18	121.8 (3)
H8B—C8—H8C	109.5	C21—C20—C19	119.6 (4)
C7—C9—H9A	109.5	C21—C20—H20	120.2
C7—C9—H9B	109.5	C19—C20—H20	120.2
H9A—C9—H9B	109.5	C20—C21—C22	121.1 (4)
C7—C9—H9C	109.5	C20—C21—H21	119.4
H9A—C9—H9C	109.5	C22—C21—H21	119.4
H9B—C9—H9C	109.5	C23—C22—C21	119.4 (4)
C7—C10—H10A	109.5	C23—C22—H22	120.3
C7—C10—H10B	109.5	C21—C22—H22	120.3
H10A—C10—H10B	109.5	C22—C23—O3	124.7 (3)
C7—C10—H10C	109.5	C22—C23—C24	119.8 (4)
H10A—C10—H10C	109.5	O3—C23—C24	115.5 (3)
H10B—C10—H10C	109.5	C19—C24—C23	121.0 (3)
C14—C11—C6	111.2 (3)	C19—C24—H24	119.5
C14—C11—C12	110.8 (3)	C23—C24—H24	119.5
C6—C11—C12	110.4 (3)	O3—C25—H25A	109.5
C14—C11—C13	107.0 (3)	O3—C25—H25B	109.5
C6—C11—C13	111.0 (3)	H25A—C25—H25B	109.5
C12—C11—C13	106.3 (3)	O3—C25—H25C	109.5
C11—C12—H12A	109.5	H25A—C25—H25C	109.5
C11—C12—H12B	109.5	H25B—C25—H25C	109.5
H12A—C12—H12B	109.5		
			
C17—N1—N2—C18	174.9 (3)	C1—C6—C11—C12	62.1 (5)
O1—C1—C2—C3	−179.3 (3)	C5—C6—C11—C13	−1.3 (5)
C6—C1—C2—C3	−1.2 (5)	C1—C6—C11—C13	179.7 (4)
O1—C1—C2—C7	0.7 (5)	C3—C4—C15—S1	111.0 (3)
C6—C1—C2—C7	178.8 (3)	C5—C4—C15—S1	−71.0 (4)
C1—C2—C3—C4	1.3 (5)	C16—S1—C15—C4	−71.7 (3)
C7—C2—C3—C4	−178.7 (3)	C15—S1—C16—C17	−71.2 (3)
C2—C3—C4—C5	−1.2 (5)	N2—N1—C17—O2	−177.0 (3)
C2—C3—C4—C15	176.9 (3)	N2—N1—C17—C16	−0.3 (5)
C3—C4—C5—C6	0.9 (5)	S1—C16—C17—O2	97.8 (4)
C15—C4—C5—C6	−177.2 (3)	S1—C16—C17—N1	−78.9 (4)
C4—C5—C6—C1	−0.8 (5)	N1—N2—C18—C19	−179.0 (3)
C4—C5—C6—C11	−179.8 (3)	N2—C18—C19—C24	−179.0 (3)
O1—C1—C6—C5	179.1 (3)	N2—C18—C19—C20	0.5 (5)
C2—C1—C6—C5	1.0 (5)	C24—C19—C20—C21	0.9 (5)
O1—C1—C6—C11	−1.8 (5)	C18—C19—C20—C21	−178.5 (3)
C2—C1—C6—C11	−180.0 (3)	C19—C20—C21—C22	−0.6 (6)
C3—C2—C7—C10	119.7 (4)	C20—C21—C22—C23	−0.3 (6)
C1—C2—C7—C10	−60.3 (5)	C21—C22—C23—O3	−179.1 (3)
C3—C2—C7—C9	−115.8 (4)	C21—C22—C23—C24	1.0 (5)
C1—C2—C7—C9	64.3 (4)	C25—O3—C23—C22	3.3 (5)
C3—C2—C7—C8	1.6 (5)	C25—O3—C23—C24	−176.8 (4)
C1—C2—C7—C8	−178.4 (3)	C20—C19—C24—C23	−0.3 (5)
C5—C6—C11—C14	117.7 (4)	C18—C19—C24—C23	179.2 (3)
C1—C6—C11—C14	−61.3 (4)	C22—C23—C24—C19	−0.7 (5)
C5—C6—C11—C12	−118.9 (4)	O3—C23—C24—C19	179.4 (3)

### Hydrogen-bond geometry (Å, °)

**Table:**

*D*—H···*A*	*D*—H	H···*A*	*D*···*A*	*D*—H···*A*
N1—H1···O2^i^	0.88	2.00	2.884 (4)	176

Symmetry codes: (i) −*x*+1, −*y*+1, −*z*+1.
